# Unusual Posterior Epidural Migration of Intervertebral Herniated Disc: A Report of Two Cases

**DOI:** 10.5704/MOJ.1811.012

**Published:** 2018-11

**Authors:** FC Tamburrelli, A Perna, MS Oliva, I Giannelli, M Genitiempo

**Affiliations:** Department of Orthopaedics, Catholic University of Sacred Heart, Rome, Italy

**Keywords:** intervertebral disc, posterior herniation, epidural space, epidural neoplasia

## Abstract

Disc herniation is one of most common causes of spine surgery. Because of the presence of posterior longitudinal ligaments, disc fragments often migrate into the ventral epidural space. A posterior epidural herniation of a disc fragment is a rare occurrence. We report two cases of posterior migrated disc fragments, with, radiological and clinical findings. Because of the rarity of a posterior migration of the intervertebral disc fragments, a differential diagnosis can be challenging. This painful syndrome associated with neurological lower limb deficits can be confused initially, with other posterior epidural space-occupying lesions such as tumours, abscess or hematomas. A gadolinium-enhanced MRI scan is the gold standard for a correct diagnosis. Early surgical decompression of the spine with a posterior approach remains the optimal technique in ensuring the best possible outcome for the patient.

## Introduction

Disc herniation is very common, representing one of the major reasons for spine surgery. Literature reviews estimate that 35 to 72% of all lumbar disc herniation cause fragment migration, usually in the anterior and anterolateral epidural space^1^, whereas only very few cases of dorsal epidural space migration occur^2-4^. We report two cases of posterior migrated disc fragments, in whom neurologic symptoms and deficits were present at the time of admission.

## Case Report

### Case 1

**Fig. 1:**
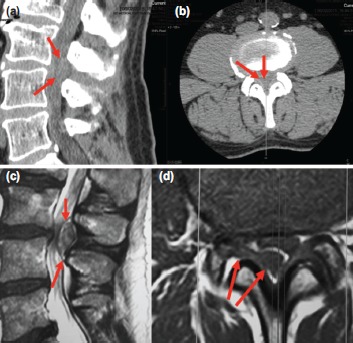
(a, b) Sagittal and axial CT-scan images that showed a ill-defined rounded lesion localised in the posterior epidural space at L3-L4 lumbar level (arrows). Diagnosis was difficult due to the low quality of definition. (c, d) Sagittal and axial images on T2-weighted MRI focused on the indexed lumbar level that clearly showed the presence of a large fragment of disc material migrated posteriorly to the dural sac that caused severe cauda equine compression.

A 53-year old man was admitted to our emergency unit with low back pain (Visual Analog Scale VAS 7), bilateral leg pain (VAS 9) and acute paraparesis with leg extension and bilateral hip flexion deficit without trauma. A neurological examination found that the strength in the right hip flexion and the right knee flexion was grade 2 with bilateral hypoesthesia in the L4 and L5 region. No other neurologic abnormalities were found. Blood tests revealed no alterations. CT scan of lumbar spine did not reveal any significant findings. Therefore, an MRI of the lumbar spine was performed. MRI images showed a mass in the dorsal epidural space with compression of the epidural sac at the L3-L4 level (Fig. 1). Fifteen hours after admission the patient underwent surgery through a posterior approach and right hemi-laminectomy at the L3 level. After the removal of the ligamentum flavum, a disc material was found, which was responsible for compression and displacement of the dural sac. A disc inspection at L3-L4 level revealed a tear of the disc's annulus. Two days after surgery the patient experienced significant pain relief (VAS back 3, VAS leg 2), and recovery of the right hip flexion and right knee flexion strength. At the one month follow-up, patient had complete neurological recovery.

### Case 2

**Fig. 2:**
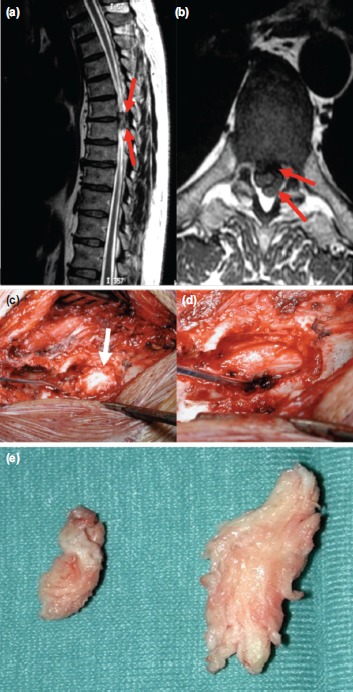
(a,b) T2-weighted MRI images of the thoracic spine that showed a large herniated disc fragment migrated posteriorly that caused cord compression. The severity of the cord compression was more evident on the axial plane. Intraoperative images after posterior laminectomy (c) before and (d) after herniated disc removal. (e) Macroscopic image of the fragment of disc surgically removed.

A 49-year old man was admitted to our emergency unit for acute dorsal pain experienced during driving. He had no trauma. The patient had bilateral hypoesthesia in the L2-L3-L4-L5 region and VAS for back pain was 9. A neurologic examination revealed a condition of paraparesis. The strength of knee flexion and extension was grade 3 bilaterally, the hip flexion was grade 3 bilaterally and the ankle flexion and extension was grade 4. The blood test revealed no alterations. MRI of the thoracic spine revealed a wide lesion in the dorsal epidural space with compression of the epidural sac at the T6-T7 level (Fig. 2 a,b). Twelve hours after admission the patient underwent decompression surgery, through a posterior approach with laminectomy at T7 level. After removing the ligamentum flavum, a large amount of disc material migrated into the dorsal epidural space was found (Fig. 2c). After the removal of herniated fragment (Fig. 2e), the dural sac showed no more signs of compression (Fig. 2d). In the first post-operative day, the patient had good pain relief (VAS back 3) and showed prompt neurological recovery. The patient was discharged five days after surgery and at the one-month follow-up evaluation he was pain-free, with no hypoesthesia or motor weakness.

## Discussion

Disc fragment migration in the spinal canal is a common condition although it generally occurs in the anterior epidural space. Posterior disc fragments migration in the epidural space is a rare condition but when it does occur, it becomes a diagnostic challenge that requires prompt diagnosis and decompression surgery. When a disc fragment is pushed back violently and crosses the posterior longitudinal ligament (PLL), posterior migration should be prevented by the presence of certain anatomic structures such as the "midline septum", the "peridural" membrane, the nerve roots and the Hoffman ligaments^4, 5^. The midline septum extends from the vertebral body to the posterior longitudinal ligament and prevents the lateral migration of disc fragments.

The peridural membrane extends from one side to the other, spanning the width of the vertebral body. Anterior dural (Hoffman) ligaments, are subtle attachments between the dura mater and the deep layer of the PLL, with two ligaments (right and left) usually present at each level. Finally, nerve roots, epidural fat and epidural venous plexus also are structures that can prevent posterior epidural migration. However, the actual role of all these structures in preventing disc fragment migration remains unknown. In this aspect, the occurrence of posterior epidural migration of disc fragments can be related to strong expulsion forces that push back the fragment over all anatomic barriers. Anatomic variations like an abnormal position of the disc, cranial or caudal to the intervertebral foramen, could be associated with a different of the position of the nerve roots and may allow for posterior migration.

The clinical presentation of these patients is characterised by significant neurological symptoms due to the compression of the nerve roots, such as cauda equina syndrome. In some cases, posterior migration is characterised by chronic back pain, while in other cases an acute onset may be observed. Diagnosis, on the other hand, can be challenging because of a variety of diseases caused by posterior epidural space-occupying lesions such as tumours, abscess or hematoma. The only way to achieve the correct differential diagnosis is a gadolinium-enhanced MRI that, in the case of extruded disc fragments due to vascularisation of the epidural fat surrounding the fragment, shows "ring enhancement” after the injection of the contrast medium^1^. Disc fragments, in 80% of cases, are generally hypointense in T1-weighted images and hyperintense on T2-weighted images. Sometimes, diagnosis is only possible during surgery where the real nature of the compression appears clearly. The treatment of choice is the removal of the extruded fragments with decompression of neurologic structures.

It should be remembered that the approach to thoracic disc herniation is usually anterior. In the second case described, a posterior approach was performed to allow a prompt spine decompression; no discectomy was performed. Early surgery had to be carried out to prevent severe neurologic deterioration and to allow neurologic recovery after surgery.

## Conflict of Interest

The authors declare no conflicts of interest.

## References

[1] Turan Y, Yilmaz T, Göçmez C (2017). Posterior epidural migration of a sequestered lumbar intervertebral disc fragment. Turk Neurosurg..

[2] Bonaroti EA, Welch WC (1998). Posterior epidural migration of an extruded lumbar disc fragment causing cauda equina syndrome. Spine (Phila Pa 1976)..

[3] Kil JS, Park JT (2017). Posterior epidural herniation of a lumbar disk fragment at L2-3 that mimicked an epidural hematoma. Korean J Spine..

[4] Dosoglu M, Is M, Gezen F, Ziyal M (2001). Posterior epidural migration of a lumbar disc fragment causing cauda equina syndrome: Case report and review of the relevant literature. Eur Spine J..

[5] Lakshmanan P, Ahuja S, Lyons K, Howes J, Davies PR (2006). Sequestrated lumbar intervertebral disc in the posterior epidural space: a report on two cases and review of the literature. Spine J..

